# The impact of medication-related osteonecrosis of the jaws on the quality of life in cancer patients

**DOI:** 10.4317/jced.56307

**Published:** 2020-08-01

**Authors:** Raquel-D’Aquino-Garcia Caminha, Patricia-Lopes Alcantara, Caroline-Gomes Carvalho, Verônica-Caroline-Brito Reia, Ana-Lucia-Alvares Capelozza, Paulo-Sérgio-da Silva Santos

**Affiliations:** 1 MSc, DDS, Department of Surgery, Stomatology, Pathology and Radiology, Bauru School of Dentistry, University of São Paulo, Bauru, Brazil; 2 DDS, Center Research Clinical, University of São Paulo, Bauru, Brazil; 3 PhD, MSc, DDS, Department of Surgery, Stomatology, Pathology and Radiology, Bauru School of Dentistry, University of São Paulo, Bauru, Brazil; 4 PhD, MSc, DDS, Department of Surgery, Stomatology, Pathology and Radiology, Bauru School of Dentistry, University of São Paulo, Bauru, Brazil

## Abstract

**Background:**

To evaluate the impact of oral health on the quality of life (QOL) of individuals undergoing cancer treatment at the time of diagnosis of medication-related osteonecrosis of the jaw (MRONJ).

**Material and Methods:**

The present cross-sectional study analyzed patients with MRONJ from 2013 to 2019. The collected data included demographic data, base disease, medications associated with MRONJ, route of administration and time of use, signs, symptoms, and tomographic features of acute MRONJ, staging according to American Association of Oral and maxillofacial Surgeons position paper 2014 (AAOMS), type of dental treatment performed, outcome, and the responses to the Oral Health Impact Profile questionnaire (OHIP-14). Statistical analysis was performed using the Tukey test to study the association between oral condition and the QOL. A p-value of less than 0.05 was considered statistically significant.

**Results:**

The sample consisted of 16 medical records of patients with MRONJ. Psychological discomfort showed alarmingly significant results (*p*< 0.001) with strong negative impact on the QOL of the patients. Functional limitation was the least affected dimension (*p* = 0.747). The other dimensions did not show statistically significant results.

**Conclusions:**

MRONJ compromises oral health and negatively impacts the QOL, especially with respect to the psychological discomfort (worry and stress). The OHIP-14 questionnaire proved to be an effective tool in the assessment of this impact.

** Key words:**Medication-related osteonecrosis of the jaw, quality of life, oral health, OHIP-14.

## Introduction

Medication-related osteonecrosis of the jaw (MRONJ) is associated with symptoms, such as halitosis, severe pain, difficulty in chewing, and dysphagia, that have a negative impact on the quality of life (QOL) (1,2). The QOL indicators are related to physical, social, and psychological well-being and can be assessed through generic or specific questionnaires according to the type of disease (3).

The Oral Health Impact Profile questionnaire (OHIP-14) (4) has been widely used (5,6) and allows the self-assessment of the individual with respect to the impact of oral health on the QOL. The OHIP-14 is a questionnaire recognized for its sensitive potential for interference in the QOL. It is a low-cost, easily applied tool, and it allows a greater access to the population. Consequently, it helps in identifying the effects on the QOL of individuals, enabling the best targeted treatment. Though studies have reported a high prevalence of MRONJ (7,8), very few have reported its impact on the QOL (5,9,10). According to the SF-12 questionnaire that evaluates the general QOL (2), MRONJ is a condition that can lead to worsening of the physical and mental state of patients. Treatment for MRONJ aims to restore the QOL by resolving the symptoms in the jaws (11).

Researchers have pointed out the scarcity in the literature about oral health impairment and the QOL of cancer patients (10,13-16). A high incidence of suicides has been demonstrated in cancer patients, probably due to the functional alterations that drastically affect the QOL (17). Therefore, the importance of preserving the QOL of these individuals through prevention and control of MRONJ (2,5,16) becomes crucial.

The aim of this study was to evaluate the impact of oral health on the QOL in individuals undergoing cancer treatment at the time of diagnosis of MRONJ.

## Material and Methods

The present cross-sectional study was conducted at a clinical research center specializing in care for systemically compromised individuals through the analysis of 902 medical records of patients who underwent dental treatment from 2013 to 2019. Sixteen medical records of patients with MRONJ were selected. This study was approved by the Research Ethics Committee of the University of São Paulo (CAAE: 65053617.0.0000.5417).

Demographic data collected from the 16 medical records included name, gender, age, base disease, medications associated with MRONJ, route of administration and time of use, signs, symptoms, and tomographic features of acute MRONJ, staging according to the norms by AAOMS (1), type of dental treatment performed, outcome of MRONJ, and impact of oral health on the QOL.

The portuguese version (18) of the OHIP-14 was used as the QOL questionnaire in which 7 dimensions were divided into 14 questions with responses attributed by the individuals through a Likert scale (0 to 4) to quantify the impact of oral health on the QOL. The value of the answers was multiplied by the power corresponding to each question to calculate the total value for each dimension.

The seven dimensions included functional limitation, physical pain, psychological discomfort, physical disability, psychological disability, social disability, and disability. Values for each dimension were calculated and the impact of each dimension was classified as low (0 - 1.33), medium (1.33 - 2.68), or high (> 2.68). According to the total values the correlation was classified as having weak impact (< 9.33), medium impact (9.33-18.66), or strong impact (> 18.66). Higher values suggested greater negative impact of oral health on the QOL of the patients. Statistical analysis of the obtained data was performed using the Tukey test to study the correlation between oral condition and the QOL. Results were considered statistically significant when the *p-value* was less than 0.05.

## Results

 Table 1,  Table 2, and  Table 3 show the demographic data and the base diseases or metastases, the clinical / imaging characteristics of patients with MRONJ, and the characteristics related to osteonecrosis-associated drugs.

**Table 1:**
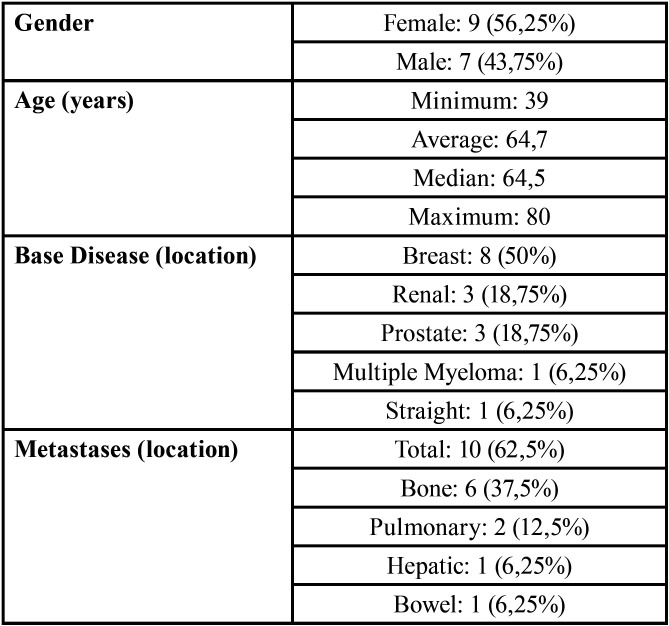
Demographic data (age, sex, type / location of cancer, and occurrence of metastasis).

**Table 2:**
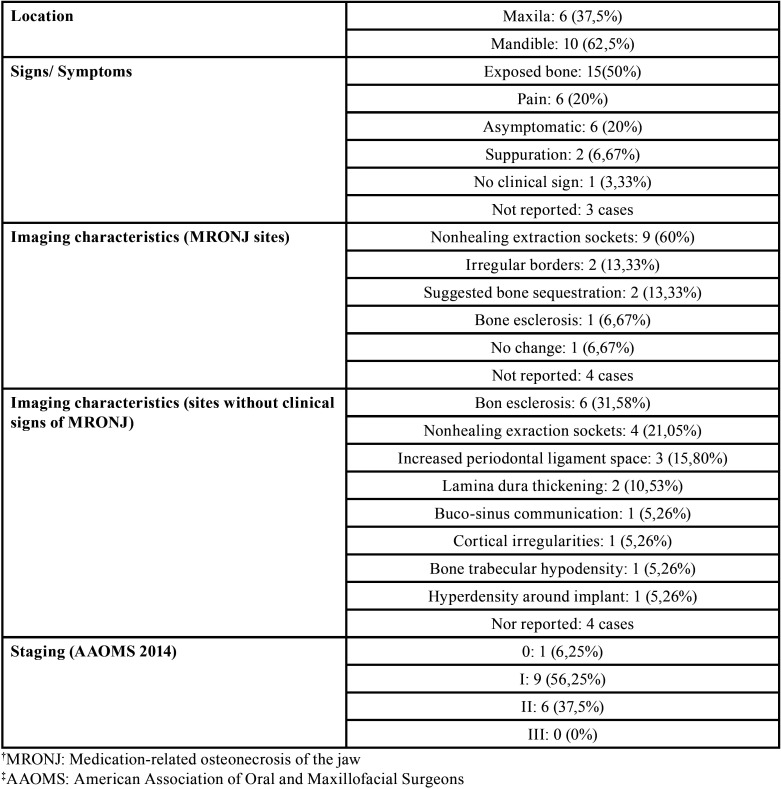
Clinical and imaging characteristics of patients with MRONJ.

**Table 3:**
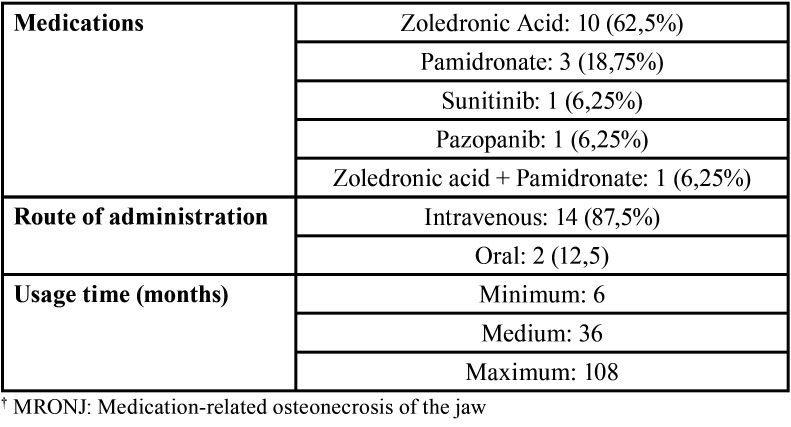
Types of MRONJ†-associated drugs.

The dimension related to psychological discomfort (D3), which evaluated the degree of concern and stress, showed the most significant (*p* < 0.001) strong negative impact on the QOL of the individuals under study. The dimension related to functional limitation (D1), which assessed the impact on word pronunciation and dysgeusia, showed the least impact (*p* = 0.747) on the QOL. No significant results were observed for the other dimensions (*p* > 0.05). The values obtained from the OHIP-14 according to the dimensions are listed in Table 4.

**Table 4:**
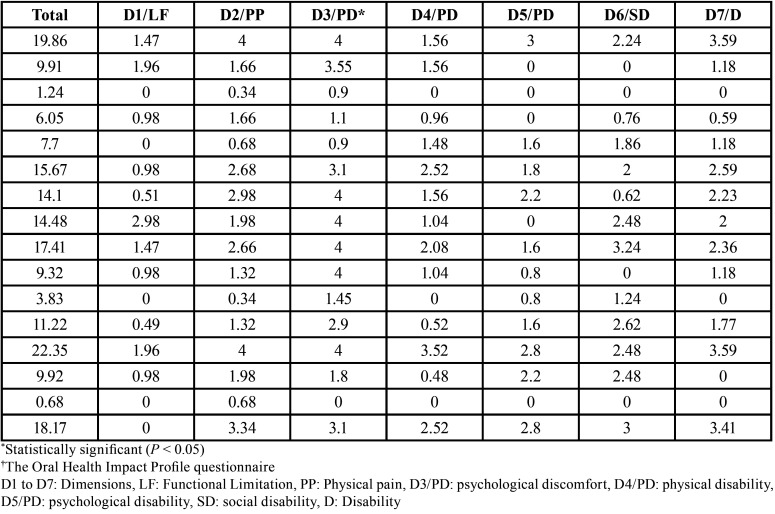
Results after application of the OHIP-14† questionnaire according to the total value and the 7 dimensions.

## Discussion

Demographic factors such as age, gender, and location of the neoplasm may interfere with the development of MRONJ. In the present study, 9 (56.25%) cases were female. The relatively higher incidence of the disease in females may be due to a higher frequency of diseases such as breast cancer and osteoporosis in women (1), and subsequently, greater indication of the drugs that may be responsible for MRONJ. Mandible was the most affected region (10 [62.5%] cases) followed by the maxilla (6 [37.5%] cases). These findings can be explained by the local vascular characteristics. Since the mandible has less vascularization when compared with the maxilla (16), the incidence of osteonecrosis may be higher.

The OHIP-14(18) questionnaires were applied at the time of the diagnosis of MRONJ. The responses showed alarming negative results for the dimension of psychological discomfort in which the highest score regarding oral health concerns and stress was observed. After comparison among the dimensions of functional limitation (D1), physical pain (D2), and psychological discomfort (D3), highest scores were seen for D3 followed by D2. The scores for the dimension D1 were the lowest when compared with all the other dimensions.

A recent study showed that the most affected dimensions were functional limitation (D1), physical pain (D2), and physical disability (D4). These findings are different from results of the present study. OHIP-14 can be applied at different stages including before, during, and after the cancer treatment. This approach allows the comparisons among various stages and may directly interfere with the results. Another study emphasized that psychological discomfort (D3) is generated by the high level of anxiety and acute pain in patients with MRONJ (19). Since the present study was a cross-sectional study, OHIP-14 was applied only after the presence of MRONJ was confirmed. This may be a limitation of the present study, since comparisons during the other stages in the same patients was not possible.

The imaging characteristics of MRONJ are more frequently observed in the more advanced stages where it is possible to observe changes such as a larger area of bone involvement, painful symptoms, and neurosensitive alterations, which result in alterations in chewing and swallowing and significant deterioration in the QOL. (20,21) 

The stagewise classification facilitates the diagnosis and the therapeutic management of MRONJ (1). Among the 4 classification stages (0, I, II, III), stage I was the most prevalent in the present study, followed by stage II and stage 0. Stage III was not observed. The treatment of MRONJ aims to reduce the painful symptoms, control infection, and reestablish social contact with family and friends, thus improving the physical and mental state and consequently improving the QOL of these individuals (11,22).

QOL was negatively affected by psychological discomfort in our study with a strong correlation (*p* < 0.001), which we highlight as a differential (5,23). Since concern and stress is associated with patients’ oral health and osteonecrosis symptoms, it is important to develop a multidisciplinary treatment approach with medical, dental, and psychological support to minimize the impact of MRONJ on the QOL.

The best way to assess the impact of oral health on the QOL would be to apply the OHIP-14 before and after the MRONJ treatment and compare the results. This approach was not possible in the present study, as some patients died due to disease complications.

The results of the present study showed that MRONJ negatively affects the oral health of the patients, especially with respect to psychological discomfort. The OHIP-14 proved to be an effective tool in assessing the impact of oral problems on the QOL.
